# Use of Sea Fennel as a Natural Ingredient of Edible Films for Extending the Shelf Life of Fresh Fish Burgers

**DOI:** 10.3390/molecules25225260

**Published:** 2020-11-11

**Authors:** Daniel Rico, Irene Albertos, Oscar Martinez-Alvarez, M. Elvira Lopez-Caballero, Ana Belen Martin-Diana

**Affiliations:** 1 Subdirection of Research and Technology, Agro-Technological Institute of Castilla y León, Consejería de Agricultura y Ganadería, Finca de Zamadueñas, Ctra. Burgos km. 119, 47171 Valladolid, Spain; mardiaan@itacyl.es; 2 Santa Teresa de Jesús Catholic University of Ávila (UCAV), Calle Canteros s/n, 05005 Ávila, Spain; irene.albertos@ucavila.es; 3 Institute of Food Science, Technology and Nutrition (ICTAN, CSIC), 10, Jose Antonio Novais, St., 28040 Madrid, Spain; oscar.martinez@ictan.csic.es (O.M.-A.); elvira.lopez@ictan.csic.es (M.E.L.-C.)

**Keywords:** sea fennel, chitosan, antioxidants, edible film, fish mince

## Abstract

The growing interest from consumers toward healthy and nutritious products and their benefits for health has increased the consumption of whole and processed fish. One of the main problems of fish is the short shelf life, especially when it is processed as in the case of burgers. The use of edible coating is an interesting strategy to extend the quality and safety of the product, reducing the need for artificial preservatives. This study evaluated the use of chitosan-based edible film formulated with sea fennel plant and sea fennel extracts. The analyses showed than the use of edible film extended the shelf life of fish burgers regardless of the incorporation of sea fennel mainly associated to the gas barrier properties and selective permeability of the film applied to the fish surface. The incorporation of sea fennel in the films did not produce any antimicrobial enhancement, although sea fennel (mostly extract) produced a better pH and enhanced the antioxidant properties and lipid oxidation of fish burgers. However, sensory analyses showed than fish burgers coated with sea fennel film plant had better acceptability than those with sea fennel extracts, probably due to the better odour and colour of the whole plant during storage. The study showed that the use of sea fennel plant at 12.5% extended the shelf life of fish burgers using a safe and clean label strategy.

## 1. Introduction

Halophilic plants are species that survive in difficult environments. Through different morphological or physiological adaptations, these plants are capable of balancing the excess salinity; some grow in dry and hard soils and others do so in humid spaces or directly flooded by seawater [1]. Many of these plants have gained popularity in medicinal and cosmetic applications, and others have been cultivated for ornamental use, particularly succulent varieties, which are highly appreciated in gardens in warm areas.

In recent years, gastronomy has started to pay attention to these forgotten plants, including them in their new recipes, and increasing their demand and availability in the retail market. Beyond their value as dish decoration, their rediscovered organoleptic characteristics give these plants an important gastronomic potential [2].

On the other hand, halophyte plants are a promising ingredient in functional food products and/or a source of bioactive (antioxidant activity) compounds, and they have great economic potential [3,4].

*Crithmum maritimun* or sea fennel is a halophyte plant located in maritime areas and sometimes in the sand in the Mediterranean Sea and Atlantic Ocean [5]. It is used in traditional recipes in countries such as Italy, Greece, Great Britain or France [6]. Traditionally, the plant serves as a condiment in salads, owing to its spicy and salty taste, or it may be pickled [7]. Leaves can be consumed fresh in salads, or cooked in soups and sauces [2]. Flower top and stalk infusion have been used as herbal teas [8]. Its seeds also contain appreciable amounts of oil, with a fatty acid composition similar to that of olive oil [7].

Recently, sea fennel has gained interest due to its nutritional and bioactive properties, which include vitamin C, iodine, carotenoids, calcium and fiber [1]. However, this halophyte is under-utilized for commercial cultivation [6]. The nutritional characteristics and phenolic profile of this plant have been characterized by the authors in a previous work [9], where its potential as a nutritive food ingredient was highlighted. Moreover, the presence of bioactive compounds, especially hydroxycinnamic acids, makes it a potential candidate for bioactive food formulation. One way to take advantage of bioactive properties of novel ingredients is the formulation of these or their extracts in edible films and coatings, which can be used for extending the shelf life of perishable food. Edible films and coatings are excellent carriers of bioactive compounds and gradually release them over storage [10,11,12].

Fish are susceptible to rapid spoilage, but due to their high nutritional value, they are a part of most diets around the world. Loss of freshness is caused by enzymatic and chemical reactions, along with microbial activity. Atlantic horse mackerel (*Trachurus trachurus*) is one of the most important pelagic fisheries in southern Europe. This species contains high levels of long-chain omega-3 polyunsaturated fatty acids (PUFAs), essential nutrients with well-known beneficial effects on human health. However, these species are under-utilized due to their high susceptibility to oxidation, which is directly related to the production of off-flavors and off-odors [13]. In the global marketplace, increasing consumer demand has resulted in longer logistic chains. Great efforts have been made to enhance the shelf life in fresh Atlantic horse mackerel.

Chitosan, along with alginate, is one of the most used polymers in edible coating formulations. Chitosan is produced from chitin by deacetylation in the presence of an alkaline base. It is a polymer, formed by units of β-(1-4)-2-acetamido-glucose and β-(1-4)-2-amino-glucose, the latter generally being more than 80%. The importance of this biopolymer lies in its antimicrobial properties together with its ability to form films [14], making it a perfect vehicle for transporting other compounds in coating formulations. It is hydrophilic, biocompatible, and biodegradable, and it is the main structural component of crab, lobster and shrimp shells. Chitosan has many specific properties such as antimicrobial activity, non-toxicity, and a remarkable affinity for proteins.

The inclusion of antioxidants or antimicrobial compounds in edible chitosan-based films [15] is a popular strategy in the industry since these compounds can enhance the antimicrobial or antioxidant capacity of chitosan, which is reduced due to its poor solubility. For this reason, the inclusion of sea fennel in edible film formulation is suggested as a strategy to extend the shelf life and enhance the nutritional value of the final product and also to promote an environmental strategy to valorize a plant with scarce use and with gastronomic and technological potential [16,17,18,19]. The objective of this study was to evaluate the effectiveness of sea fennel plant and extract in a chitosan-based edible film for extending the shelf life of fish burgers.

## 2. Results and Discussion

### 2.1. Headspace Gas Analysis

**Figure 1:**
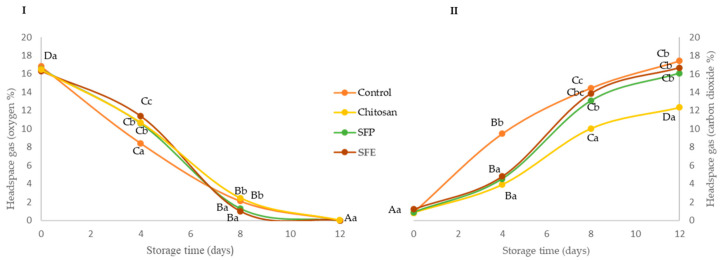
Oxygen (**I**) and carbon dioxide (**II**) headspace gas concentration (%) of Atlantic horse mackerel burgers coated with different film formulations (control without coating film, chitosan, sea fennel plant (SFP) incorporated in a chitosan-based film and sea fennel extract (SFE) incorporated in a chitosan-based film) and stored at 4 °C for 12 days. Different uppercase letters indicate significant differences (*p* < 0.05) over storage time in each treatment. Different lowercase letters indicate significant differences (*p* < 0.05) due to the treatment.

Changes in O_2_ and CO_2_ concentration were evaluated in fish burgers coated with edible films during storage at 4 °C (Figure 1). Edible films reduced the exchange of O_2_ and CO_2_ between the burger and the surrounding atmosphere, mainly at the beginning of storage compared to the control. In the case of oxygen (Figure 1I), no significant (*p* ≥ 0.05) differences were observed between burgers coated regardless of the incorporation of sea fennel. At day 8, a significant (*p* ≤ 0.05) depletion of oxygen was observed in all the samples, and the packaged coated burgers had similar O_2_ concentrations (2–4%), meanwhile the control burgers reached oxygen values close to zero. At the end of the storage (12 days), all fish burgers showed O_2_ values close to zero. These lower O_2_ concentrations might be associated with lipid oxidation [20] and the growth of aerobic bacteria [21]. In the case of CO_2_ (Figure 1II), a significantly higher increase was observed at day 4 in the control samples, as compared with the coated ones. These lower CO_2_ levels may be due to the good gas barrier properties of the chitosan films [22]. The barrier properties of polymer-only films, as is the case with chitosan film without sea fennel, may have been altered by the introduction of the sea fennel plant or extract, and the capacity to retain microbial CO_2_ production was diminished, as observed from day 8.

The study showed that the incorporation of sea fennel on the formulation did not produce any enhancement on the headspace during the storage, probably due to internal gas atmosphere modification, which might only be associated with the gas barrier properties and selective permeability of the chitosan applied to the fish burger surface, and their association with relative humidity and temperature will have an important effect on the changes in endogenous O_2_ and CO_2_ levels.

### 2.2. pH

The pH of fish burgers increased over storage time (Table 1) in all treatments. The initial values (6.44–6.82) were similar to values reported by other authors [23,24] in minced horse mackerel. This relatively high pH (pH > 6) facilitates the microbial spoilage in fish [21]. The pH values were lower in burgers coated with edible films compared to control until day 8 when all of them reached similar values regardless of the coated film. The edible films probably avoid pH increases by preventing protein and nucleotide degradation and subsequent alkaline by-product liberation [25] during the first days of storage. The addition of sea fennel produced a significant control of pH, with lower changes in those samples coated with chitosan films, probably due to the antimicrobial activity of sea fennel plant and extract, which controlled the antimicrobial and oxidative processes of the burgers. Fish burger coated only with chitosan showed intermediate values.

### 2.3. Water Activity (a_w_)

Water activity plays an important role in fish spoilage and the growth of microorganisms. In this sense, the effect of a_w_ on the different treatments was studied and the relation between a_w_ and other spoilage indicators established. As a general pattern, the use of edible films reduced the a_w_ of burgers until day 8 of storage (Table 2). This decrease in the a_w_ could be related to a reduction of the microbial counts in the samples with films (Chitosan, SFP and SFE). Among edible films, fish burger coated with SFE showed the lowest a_w_ over storage time, which could be related to the higher antimicrobial activity of the extract in the edible film formulated with the whole plant (SFP).

### 2.4. Moisture Content

The behavior of the moisture content of the fish samples over storage time followed different trends in samples with or without edible film (Table 3). Edible films reduced the initial moisture content, probably due to water absorption. This behavior may favor microbial control. The effect of retaining moisture may have been overridden over time and surrounding moisture gained, with samples showing similar moisture content at the end of the storage, with no differences among treatments (control, chitosan, SPE, SFE) at day 12. Nevertheless, a protective effect on the samples biochemical and microbiological stability may have been exerted by the use of edible films, although no significant differences due to the incorporation of sea fennel were observed for this parameter.

### 2.5. Microbiological Analysis

One of the major problems of fish burgers is spoilage during storage, which causes a short shelf life. It is essential to control growth and extend the product storage time.

**Figure 2:**
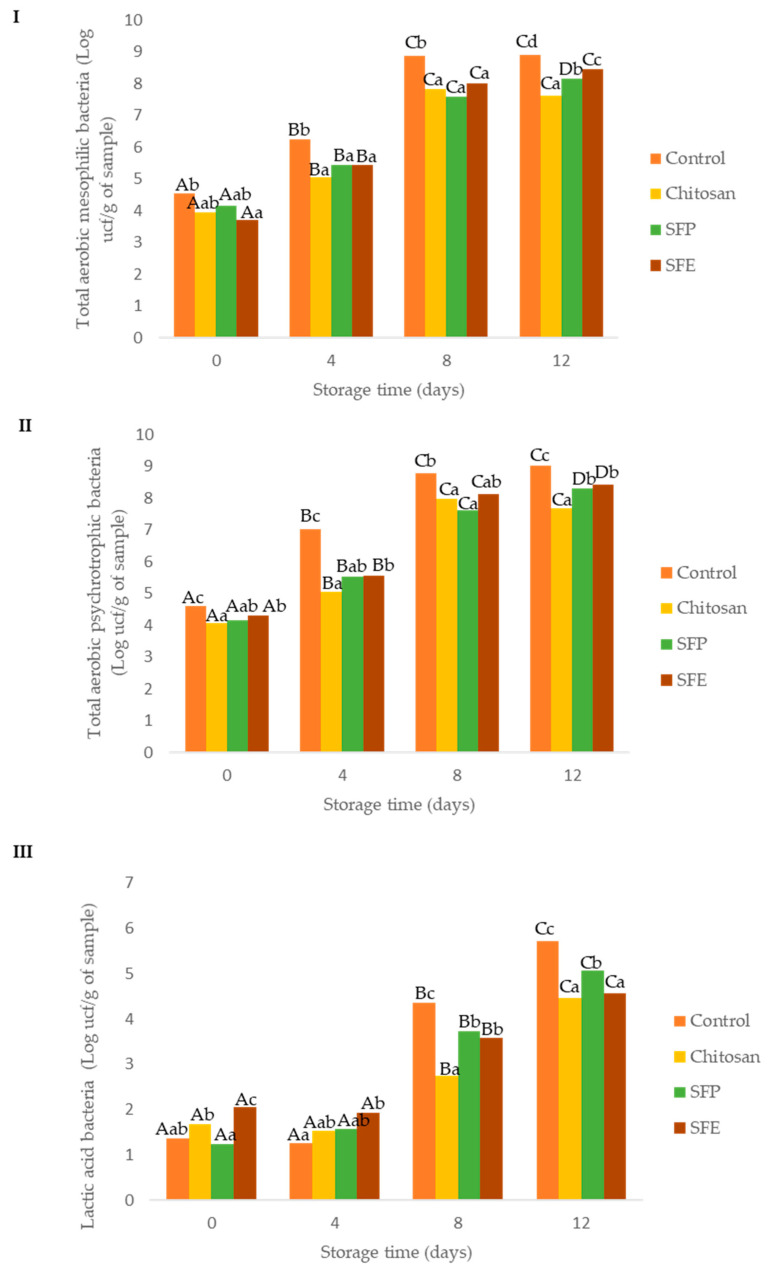
Effect of edible films on the growth of total aerobic mesophilic (**I**), total aerobic psychrotrophic (**II**) and lactic acid (**III**) bacteria in Atlantic horse mackerel burgers coated with different film formulations (control without coating film, chitosan, sea fennel plant (SFP) incorporated in a chitosan-based film and sea fennel extract (SFE) incorporated in a chitosan-based film) and stored at 4 °C for 12 days. Different uppercase letters indicate significant differences (*p* < 0.05) over storage in each treatment. Different lowercase letters indicate significant differences (*p* < 0.05) due to the treatment.

Initially, total aerobic mesophilic bacteria ranged between 3.70 and 4.53 log CFU per g, which was similar to Alfaro and Hernández [26], who studied Atlantic horse mackerel fillets. Control fish burgers showed higher total aerobic mesophilic bacterium counts over storage time, as compared to the burgers coated with edible films (Figure 2I). At day 4, control samples exceeded the values recommended for saleability (10^6^ CFU per g), while coated samples were below this level up to day 4, but exceeded the limit by day 8 [27]. Edible films exerted a significant protective role, increasing the shelf life from 4 to 8 days.

Total aerobic psychrotrophic bacteria counts in Atlantic horse mackerel burgers were at similar levels to those of total aerobic mesophilic counts (Figure 2II). This was expected, as the microflora of fish is dominated by psychrotrophic species [21]. These higher microbial load reductions in the samples where the film was used could be also explained by other physicochemical parameters (a_w_ and pH). Water activity (a_w_), an important factor for microbial growth, was lower in burgers covered with film (chitosan, SFP, SFP) when compared to no film (control). Control samples also presented higher pH values due to alkaline compounds, which are formed from protein and nucleotide decomposition in the muscle during the post-mortem period [25].

Lactic acid bacteria (LAB, Figure 2III) were also evaluated as part of the natural microflora of Atlantic horse mackerel burgers. The initial counts of LAB were below 5 log CFU per g up to 4 days of storage. The headspace composition (O_2_ and CO_2_) can explain this fact. At day 4, control samples had higher CO_2_ and lower O_2_ concentrations (Figure 1) than those found in film-covered samples (chitosan, SFE and SFP). This atmosphere may benefit LAB growth, which are facultative aerobic microorganisms. After day 4, headspace composition of the control (control) became similar to that of the rest of the samples (chitosan, SFE and SFP), but the control still showed higher LAB counts at days 8 and 12, following a similar trend as the results observed for the other two groups of bacteria evaluated (total aerobic mesophilic bacteria and total aerobic psychrotrophic bacteria).

Microbial growth is the major cause of fish spoilage and its metabolism resulting in the formation of amines, sulphides, alcohols, ketones and organic acids with unpleasant and unacceptable off-flavors [28]. The antimicrobial effect of chitosan films has been widely described in the literature. The antimicrobial mechanism of chitosan is due to its positive charge, which may compete with Ca^2+^ ions for the negatively charged bacterial membrane [29]. A previous work [30] also demonstrated that chitosan films reduced microbial spoilage (total aerobic mesophilic, lactic acid bacteria and total coliform) in trout fillets.

Edible films with sea fennel (SFE, SFP) did not significantly improve the antimicrobial activity in comparison with chitosan-only films (chitosan). On the other hand, Jallali et al. [31] demonstrated that sea fennel essential oil and acetonic extracts had antimicrobial activity in an in vitro test against *E. coli*, *p. aeruginosa* and *S. aureus*. Essential oils showed the best antimicrobial activity due to the presence of oxygenated monoterpenes. The low antimicrobial activity of the extract used in the edible films (SFE) can be attributed to poor water extraction; optimization of the solvent-pressure selection may improve the already significant antimicrobial activity observed [32].

### 2.6. Determination of Malondialdehyde (MDA)

Atlantic horse mackerel muscle tissue has a high susceptibility to oxidation because it contains large amounts of hemoglobin (pro-oxidant) and polyunsaturated fatty acids [33]. Furthermore, the mincing process rapidly affects lipid oxidation due to cellular disruption, exposition of the flesh to air, and subsequent activation of lipoxygenases, which can initiate polyunsaturated fatty acid oxidation [34].

**Figure 3:**
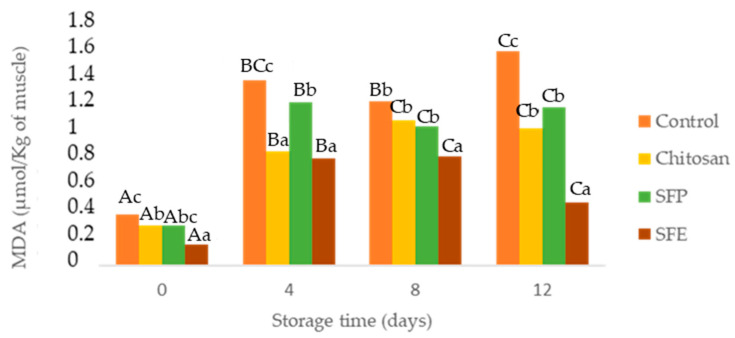
Determination of malondialdehyde (MDA) in Atlantic horse mackerel coated with different film formulations (control without coating film, chitosan, sea fennel plant (SFP) incorporated in a chitosan-based film and sea fennel extract (SFE) incorporated in a chitosan-based film) and stored at 4 °C for 12 days. Different uppercase letters indicate significant differences (*p* < 0.05) over storage time in each treatment. Different lowercase letters indicate significant differences (*p* < 0.05) due to the treatment.

Secondary lipid oxidation was monitored through thiobarbituric acid reactive substances. As expected, MDA (Figure 3) showed a significant increase over storage. However, these values were maintained below the limit beyond which fish would normally develop undesirable odors (1–2 µg MDA/g) [35]. All edible films (chitosan, SFP, SFE) were effective in comparison with the control for reducing lipid oxidation. Sea fennel is rich in antioxidant compounds such as vitamin C, carotenoids and phenolics [7]. The most common phenolic compounds detected in sea fennel include phenolic acids, chlorogenic acid, neochlorogenic, cryptochlorogenic, ferulic acid and caffeoylquinic acids and its derivatives. Gallic, caffeic, vanillic, rosmarinic and p-coumaric acids have been also previously reported in this halophyte [9,31].

These results could also be explained based on lower oxygen contact with the samples covered by chitosan films and gas barrier properties [36]. Among treatments, films with sea fennel extract (SFE) prevented lipid oxidation more than plant (SFP) or chitosan-only films (Chitosan). In [37], the authors reported similar results with seaweed extracts, which better-prevented lipid oxidation in fish burgers when edible films were applied directly on the fish samples.

### 2.7. Antioxidant Capacity

**Figure 4:**
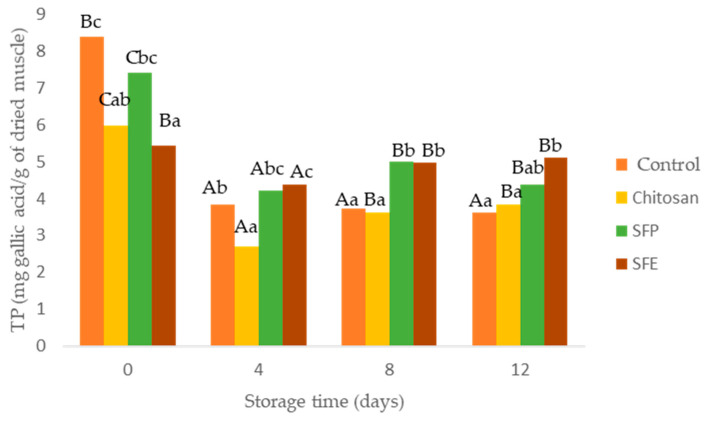
Total phenol content (TP) in Atlantic horse mackerel burgers coated with different film formulations (control without coating film, chitosan, sea fennel plant (SFP) incorporated in a chitosan-based film and sea fennel extract (SFE) incorporated in a chitosan-based film) and stored at 4 °C for 12 days. Different uppercase letters indicate significant differences (*p* < 0.05) over storage time in each treatment. Different lowercase letters indicate significant differences (*p* < 0.05) due to the treatment.

The effectiveness of edible films as antioxidants was evaluated using total phenol content (TP) and oxygen radical absorbance (ORAC). A decrease in TP determined in burger samples was observed over storage time (Figure 4). This decrease was in accordance with increasing lipid oxidation (MDA). The films formulated with sea fennel (SFP, SFE), especially SFE, significantly prevented the loss of TP in Atlantic horse mackerel burgers over storage time. Chlorogenic acid, a hydroxycinnamic acid, is the most abundant phenolic compound detected in sea fennel aqueous extract [8,9,38]. Sea fennel had relatively high phenolic content compared to other crops species such as different vegetables (spinach, broccoli). Total phenol content depends on the growing season and the place where sea fennel grows. Sandhill plants accumulated more TP, namely chlorogenic acid, than those growing on cliffs [38].

**Figure 5:**
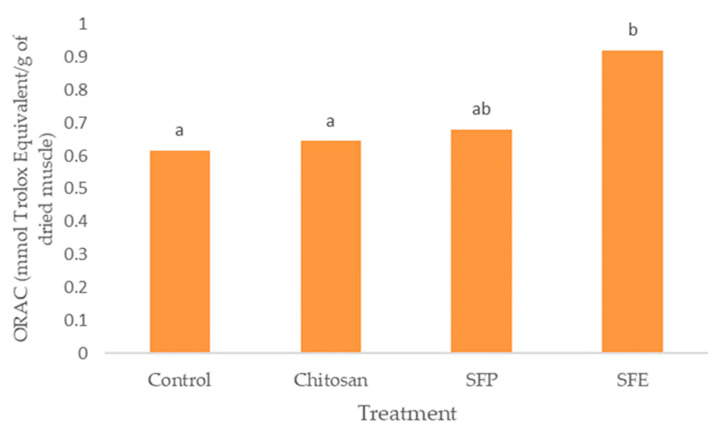
Effect of edible films on oxygen radical absorbance capacity (ORAC) in Atlantic horse mackerel coated with different film formulations (control without coating film, chitosan, sea fennel plant (SFP) incorporated in a chitosan-based film and sea fennel extract (SFE) incorporated in a chitosan-based film) and stored at 4 °C for 12 days. Different lowercase letters indicate significant differences (*p* < 0.05) due to the treatment.

Burgers covered with sea fennel extract (SFE) also showed the highest antioxidant activity measured by ORAC (Figure 5). Edible films with the plant (SFP) did not differ from chitosan films (chitosan). However, the use of edible films was effective in increasing the antioxidant activity of the samples.

SFE displayed high total antioxidant activity due to the presence of phytochemicals such as phenolic compounds. These results showed a similar trend between TP and ORAC. Hydroxycinnamic acids, such as chlorogenic acid, demonstrated that the high capacity for donating electrons is the main mechanism for retarding the development of rancidity in fish muscle [39]. Siracusa et al. [8] also attributed the antioxidant activity of sea fennel aqueous extract to the dominant presence of chlorogenic acid and of which the antioxidant properties are well-known. Sea fennel antioxidant activity was comparable with that of commercial antioxidants (BHT and BHA) according to Siracusa et al. [8].

### 2.8. Color

The color values were expressed as lightness (L*), redness (a*) and yellowness (b*). The addition of plant and/or extract of sea fennel (SP, SFE) diminished the lightness (L*) of the burgers (Table 4). This increase was more noticeable in burgers coated with SFP. In all treatments (control, chitosan, SFE), lightness (L*) significantly rose over storage time except for burgers with plant edible films (SFP) where L* values were steady. This reduction in the L* parameter can be attributed to the discoloration of the fish muscle [40].

**Figure 6:**
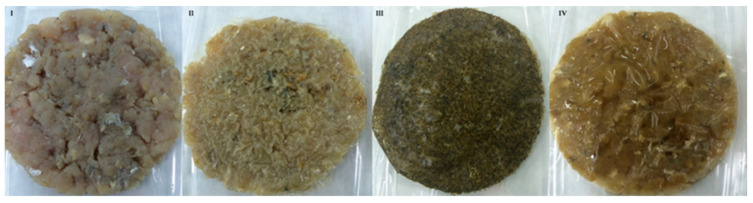
Fish burger from Atlantic horse mackerel coated with different film formulations (control without coating film—**I**, chitosan—**II**, sea fennel plant (SFP)—**III** incorporated in a chitosan-based film and sea fennel extract (SFE) incorporated in a chitosan-based film—**IV**).

Redness (a*) and yellowness (b*) values did not provide information regarding oxidation changes. Color modifications are caused by the migration of sea fennel components from the film to the fish. Burgers with edible film with SFP and SFE had higher a* and b* values in comparison with control and chitosan films over storage. Hue and Chroma were significantly different to fish burgers coated with chitosan or without film (control) and both parameters were modified during storage as a consequence of oxidation processes that affect the color of fish burgers. As shown from the color results, application of the films significantly affected the visual aspect of the Atlantic horse mackerel burgers (Figure 6).

### 2.9. Sensorial Analysis

**Figure 7:**
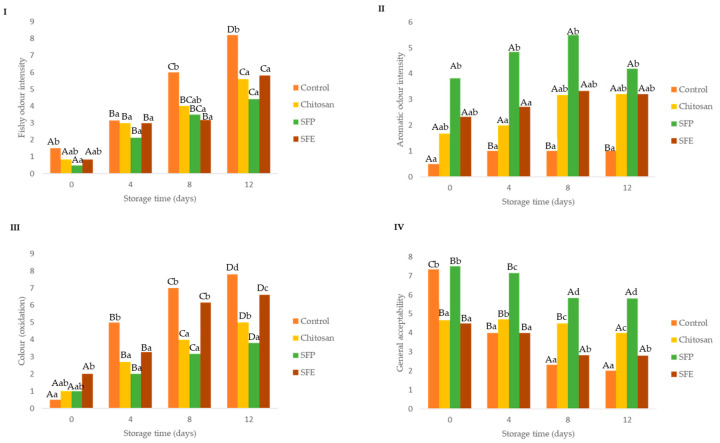
Effect of edible film sensory parameters: fishy odor intensity (**I**), aromatic odor intensity (**II**), color (oxidation) (**III**) and general acceptability (**IV**) in Atlantic horse mackerel burgers coated with different film formulations (control without coating film, chitosan, sea fennel plant (SFP) incorporated in a chitosan-based film and sea fennel extract (SFE) incorporated in a chitosan-based film) and stored at 4 °C for 12 days. Different uppercase letters indicate significant differences (*p* < 0.05) over storage time in each treatment. Different lowercase letters indicate significant differences (*p* < 0.05) due to the treatment.

Sensory parameters are important for detecting the initial quality deterioration of fish (Figure 7). Off-odor appeared and led to the rejection of fish [21]. In the descriptive analysis, results showed that the use of edible SFP and SFE delayed the appearance of fishy (off-odors, putrefaction) odor (Figure 7I) mainly from day 8 until the end of storage compared to the uncoated fish burger (control). The off odors are related to the microbial spoilage of marine fish, which is characterized by the development of fishy, rotten and H_2_S-off-odors. The spoilage developing in aerobically stored fish typically consists of Gram-negative psychrotrophic bacteria [21]. The psychotropic bacteria count agreed with the fishy odor scorings (Figure 7I).

The inclusion in the films of sea fennel plant (SFP) was perceived by the sensorial panel as an aromatic odor from the beginning of the storage until the end of the storage time (Figure 7II). This aromatic property is why this plant is used as a flavoring in many cases [13]. In contrast, the use of the extract (SFE) was not detected by the panelists at any of the times where it was analyzed.

Burgers covered with edible films with sea fennel plant (SFP) maintained their initial color better than the burgers coated with SFE or chitosan (Figure 7III). Nevertheless, this sensorial perception was not supported by oxidation analysis (Figure 4) and instrumental color measurement (Table 4). The loss of redness and increase in brown color is related to oxidation of heme proteins, hemoglobin and myoglobin.

Regarding the general acceptability (Figure 7IV), burgers covered with sea fennel plant (SEP) had higher scores over storage time, which was probably associated with the aromatic odor detected.

Panelists were asked to rank samples according to odor intensity, off-odors, color, drip loss and acceptability (Supplementary Figure S1I,II). Control fish burgers showed higher off-odors at the beginning and at the end of storage than samples coated with films, and those with sea fennel had the lowest rank values, which might be associated with higher freshness. On the other hand, the fish burgers with sea fennel in the film formulations had higher aromatic odor, which could be associated with the volatile compounds of sea fennel; an effect that was more intense with films formulated with the whole plant (SFP). Important modifications in color were associated with fish burgers with sea fennel plant and extract, and these changes are probably associated with the color provided by the plant rather than by oxidative processes. The general acceptability was higher in fish burgers coated with whole plant sea fennel (SFP).

## 3. Materials and Methods

### 3.1. Raw Material

Sea fennel (*Crithmum maritimun*) was provided by Porto Muiños S.L. The wild sea plant was harvested from the north coast of Galicia, northwest of Spain. The plants before use were sanitized using chlorinated deionized water to remove any particle or residue and immediately after were dried at room temperature.

Atlantic horse mackerel (*Trachurus trachurus*) was provided by Lonja de Burriana (Valencia, Spain), and delivered at the food processing laboratory in refrigerated polystyrene boxes 24 h post-catch. Fish was stored at 4 °C before processing and immediately processed.

### 3.2. Edible Coating Formulations

Edible sea fennel films were prepared using the whole plant and plant extract on a chitosan-based formula. The phenolic profile of this sea fennel batch was previously characterized and published elsewhere [9]. Sea fennel edible plant (SFP) were prepared using a based aqueous solution of 1.5% (*w*/*v*) of chitosan dissolved into 1% (*w*/*v*) of acetic acid. The mixture was stirred at 40 °C using an enzymatic digester (GDE, Velp Scientifica, Italy) for 2 h to obtain a homogenous solution. Afterwards, sea fennel plant was pieced in small particles (12.5% *w*/*v*) and added directly to the mixture, and 0.5 g glycerol per g of biopolymer as a plasticizer for 2 h was added to achieve complete dispersion. The films were obtained by casting 20 mL into 90 mm-diameter glass dishes and dried at room temperature for 24 h, assuring a surface density of solids in the dry films. Before analyses, the films were conditioned in desiccators over a saturated solution of KBr (58% relative humidity) at 20 ± 2 °C and relative humidity (RH) aprox. 55%.

The edible sea fennel plant extract (SFE) was prepared by homogenizing the plant on distilled water (12.5% *w*/*v*) for 1 h by stirring at 95 °C. The extraction conditions were in accordance with the optimal condition described in the UK Patent Application GB 250,667 [41]. The mixture was centrifuged and the supernatant was separated. The pellet was re-dissolved in distilled water at 95 °C with stirring for 1 h and afterwards centrifuged to obtain a second supernatant. Then, both extracts were collected and edible films were prepared by adding these extracts following the same methodology described for SFP. Edible films without sea fennel were produced to evaluate the effect of the chitosan.

### 3.3. Atlantic Horse Processing and Fish Burger Preparation

Atlantic horse mackerel (*Trachurus trachurus*) was manually beheaded, eviscerated, skinned and washed and left to drain. The skinned fishes were filleted and deboned manually in ice, and mechanically minced using a blender with a 7 mm exit pore (Lacor 69067, Guipúzcoa, Spain). Burgers of 50 g were prepared manually with a round-shaped mold to ensure all had equal size and the assay was reproducible. Four random burger batches were prepared for the different edible film treatments: control burger (without edible film), chitosan edible film burgers (chitosan), edible film sea fennel plant (SEP) and edible film sea fennel extract (SFE).

### 3.4. Coating Application and Experimental Setup

Edible films were placed on the surface of the burger, ensuring that all the fish burger was adequately covered. Afterwards, burgers were packaged in CEPT trays and sealed with a bilayer transparent film (APET/PET) with an oxygen permeability of 56 mL/m^2^/24 h/bar and water vapor transmission of 13 g/m^2^/24 h/bar.

All samples were stored at 4 °C. The assay was replicated twice and all analyses were performed in triplicate. Quality parameters were analyzed to evaluate the effectiveness of sea fennel (plant or extract) maintaining the quality of the fish burgers.

### 3.5. Quality Parameters

#### 3.5.1. Headspace Gas Analysis

Gas sampling (flow rate of 40 mL min^−1^) was carried out over 12 days using a needle connected to a mobile gas analyzer Oxybaby of Wittgas (Witten, Germany). Oxygen and carbon dioxide was monitored. To avoid modifications in the headspace gas composition due to gas sampling, each package was used only for a single measurement. Gas values were expressed as a percentage.

#### 3.5.2. pH

Ten grams of the fish burger was homogenized in 100 mL of distilled water and the mixture was filtered. The pH (pH-meter model 507, Crison, Barcelona, Spain) was measured at room temperature.

#### 3.5.3. Water Activity (a_w_)

The water activity of the fish burgers was measured with an Aqualab 4TE water activity meter (Decagon Devices Inc, Pullman, WA, USA).

#### 3.5.4. Moisture

Moisture content was gravimetrically determined [42].

#### 3.5.5. Microbiological Analysis

For microbial analysis, 10 g of the fish burgers were aseptically transferred into bags (Microgen, Surrey, UK) in 90 mL of sterile buffered peptone water and then homogenized with a pulsifier for 90 s (Pul 100E, Microgen, Surrey, UK). For each sample, appropriate serial decimal dilutions were prepared in buffered peptone water solution (1%) for the following microorganism counts:

(i) Total aerobic mesophilic count was determined using plate count agar (PCA) after incubation at 30 °C for 72 h.
(ii) Total psychrotrophic bacteria on PCA spread plates, incubated at 4 °C for 10 days.
(iii) Lactic acid bacteria (LAB) on double-layer Man Rogosa Sharpe medium incubated at 30 °C for 72 h.

#### 3.5.6. Determination of Malondialdehyde (MDA)

Lipid oxidation was measured through the analysis of malondialdehyde (MDA) using the method described by Vyncke [43]. Samples were dissolved on a 5% trichloracetic acid extract of the restructured fish muscle. A 1200 Series HPLC system (Agilent Technologies, Palo Alto, CA, USA) equipped with a quaternary pump, degasser, autosampler and a diode array detector (DAD) was used for the quantification of malondialdehyde (MDA). A C_18_ reverse-phase analytical column (150 mm × 4.6 mm internal diameter; particle size, 5 μm) (Teknokroma Analitica S.A., Barcelona, Spain) was used for the separation and held at 30 °C. The mobile phase consisting of 50 mM of KH_2_PO_4_ buffer solution, methanol and acetonitrile (72: 17:11 *v*/*v*/*v* pH 5.3) was pumped isocratically at 1 mL min^−1^. The reaction mixture was measured at 560 nm. For quantification, a curve of 1,1,3,3-tetraetoxipropane (TEP) was prepared. Results were expressed as mg of MDA per kg of dry sample.

#### 3.5.7. Total Phenols (TPs)

Fish extracts were used for TP evaluation, following the method described by Sánchez-Alonso, et al. [9]. TPs were measured on the extracts using Folin-Ciocalteu reagent, according to the Slinkard and Singleton [44] methodology, with modifications [45]. The absorbance was measured at 765 nm with a microplate reader (Fluostar Omega, BMG, Ortenberg, Germany). Results were expressed as gallic acid equivalents (GAE)/g of dry sample.

#### 3.5.8. ORAC Assay

The procedure was based on a previously reported method with slight modifications [46]. The standard curve of Trolox (15–240 mM) and samples were diluted in phosphate buffer (10 mM, pH 7.4). A volume of 150 μL fluorescein was placed in 96-well black polystyrene plates, and 25 μL of Trolox standard, sample or phosphate buffer as blank were added, all in duplicates. Samples, standards and blanks were incubated with fluorescein at 37 °C for 3 min before 2,2′-azobis (2-methyl propionamidine) dihydrochloride solution was added to initiate the oxidation reaction. Fluorescence was monitored over 35 min with a microplate reader (Fluostar Omega, BMG Ortenberg, Germany), using 485 nm excitation and 528 nm emission filters. Results were calculated using the areas under the fluorescein decay curves, between the blank and the sample, and expressed as µmol Eq. Trolox/g of dry sample.

#### 3.5.9. Color

Color parameters (lightness L*, redness a* and yellowness b*) were measured using a spectrophotometer (Minolta CM-2002, Osaka, Japan). The illuminant was D65 and 10° standard observers. Measurements were taken on burgers with edible films at eight different points to screen the entire surface.

#### 3.5.10. Sensorial Analysis

A panel of 12 panelists with previous experience in sensory analysis, aged between 20 and 45 years old, were recruited from ITACyL Staff. The training was carried out in common sessions, using a range of commercial fish burger products to establish consensus on the descriptors.

Fishy (off-odors, putrefaction) odor intensity, aromatic odor intensity, drip loss, color without film (oxidation) and general acceptability descriptors were evaluated using a 9-point scale, with 1 = minimum and 9 = maximum intensity. In addition, a ranking test was carried out to evaluate the preference of the panel. The ranking test was also valuated in the samples and the judges ranked the samples according to odor intensity, off-odors, color (oxidation), drip loss and general acceptability. Each judge was presented with a complete set of samples with random 3-digit codes for evaluation, generating one vector of multiple dependent data as shown by Carabante and Prinyawiwatkul [47].

#### 3.5.11. Statistical Analysis

Data were analyzed by MANOVA. Fisher LSD (Least Significant Difference) test was applied for determining group differences at 95% significance level. Statgraphics Centurion XVI was used for carrying out the statistical analysis.

## 4. Conclusions

There is an important need to find alternative strategies to extend the shelf life of perishable products such as fish and especially fish burgers. Edible films showed an excellent strategy to prevent microbial spoilage during storage, increasing the durability of the fish burgers three-fold.

Halophyte plants are underused by the food industry, although their composition and nutritional properties make them excellent preservative ingredients to extend food products, mostly if they are included in edible films.

Sea fennel produced a better control of oxidation, probably due to the high polyphenol content and antioxidant activity of this plant, although no further benefit on microbial spoilage was observed as compared to chitosan film alone. Although oxidative processes were controlled better with edible films formulated with extracts (SFE), the panelists ranked those samples coated with the whole plant (SFP) better, probably due to its aromatic properties.

## Figures and Tables

**Table 1:** pH of Atlantic horse mackerel burgers coated with different film formulations (control without coating film, chitosan, sea fennel plant (SFP) incorporated in a chitosan-based film and sea fennel extract (SFE) incorporated in a chitosan-based film) and stored at 4 °C for 12 days.

	Storage (Days)			
Edible Coating	0	4	8	12
Control	^A^ 6.82 ± 0.09 ^b^	^B^ 7.16 ± 0.01 ^d^	^C^ 7.27 ± 0.01 ^b^	^C^ 7.34 ± 0.00 ^c^
Chitosan	^A^ 6.47 ± 0.01 ^a^	^C^ 6.98 ± 0.06 ^c^	^B^ 6.77 ± 0.02 ^a^	^C^ 6.95 ± 0.03 ^ab^
SFP	^A^ 6.44 ± 0.01 ^a^	^A^ 6.63 ± 0.00 ^a^	^A^ 6.87 ± 0.31 ^ab^	^A^ 7.05 ± 0.00 ^b^
SFE	^A^ 6.76 ± 0.04 ^b^	^A^ 6.80 ± 0.05 ^b^	^A^ 6.89 ± 0.00 ^ab^	^B^ 6.89 ± 0.02 ^a^

Values (mean ± standard deviation, *n* = 3) followed by a different uppercase letter in the same row are significantly different (*p* < 0.05). Values (mean ± standard deviation, *n* = 3) followed by a different lowercase letter in the same column are significantly different (*p* < 0.05).

**Table 2:** Water activity (a_w_) of Atlantic horse mackerel burgers coated with different film formulations (control without coating film, chitosan, sea fennel plant (SFP) incorporated in a chitosan-based film and sea fennel extract (SFE) incorporated in a chitosan-based film) and stored at 4 °C for 12 days.

	Storage (Days)			
Edible Coating	0	4	8	12
Control	^A^ 0.9928 ± 0.0002 ^c^	^A^ 0.9916 ± 0.0009 ^b^	^A^ 0.9928 ± 0.0028 ^c^	^A^ 0.9855 ± 0.0037 ^a^
Chitosan	^A^ 0.9907 ± 0.0003 ^b^	^A^ 0.9835 ± 0.0017 ^a^	^A^ 0.9888 ± 0.0001 ^ab^	^A^ 0.9875 ± 0.0048 ^a^
SFP	^A^ 0.9901 ± 0.0007 ^b^	^A^ 0.9901 ± 0.0003 ^b^	^A^ 0.9894 ± 0.0015 ^b^	^A^ 0.9905 ± 0.0016 ^a^
SFE	^A^ 0.9869 ± 0.0011 ^a^	^A^ 0.9814 ± 0.0031 ^a^	^A^ 0.9859 ± 0.0000 ^a^	^A^ 0.9875 ± 0.0033 ^a^

Values (mean ± standard deviation, *n* = 3) followed by a different uppercase letter in the same row are significantly different (*p* < 0.05). Values (mean ± standard deviation, *n* = 3) followed by a different lowercase letter in the same column are significantly different (*p* < 0.05).

**Table 3:** Moisture content (%) of Atlantic horse mackerel burgers coated with different film formulations (control without coating film, chitosan, sea fennel plant (SFP) incorporated in a chitosan-based film and sea fennel extract (SFE) incorporated in a chitosan-based film) and stored at 4 °C for 12 days.

	Storage (Days)			
Edible Coating	0	4	8	12
Control	^A^ 77.27 ± 2.08 ^b^	^A^ 74.56 ± 0.75 ^b^	^A^ 74.69 ± 0.35 ^b^	^A^ 73.69 ± 2.04 ^a^
Chitosan	^B^ 74.16 ± 0.47 ^ab^	^A^ 72.67 ± 0.54 ^a^	^B^ 74.13 ± 0.25 ^b^	^AB^ 73.70 ± 0.37 ^a^
SFP	^A^ 71.90 ± 0.21 ^a^	^A^ 71.72 ± 0.46 ^a^	^B^ 72.86 ± 0.08 ^a^	^C^ 73.73 ± 0.00 ^a^
SFE	^A^ 72.61 ± 0.74 ^a^	^A^ 72.67 ± 0.45 ^a^	^A^ 72.93 ± 0.21 ^a^	^A^ 73.29 ± 0.40 ^a^

Values (mean ± standard deviation, *n* = 3) followed by a different uppercase letter in the same row are significantly different (*p* < 0.05). Values (mean ± standard deviation, *n* = 3) followed by a different lowercase letter in the same column are significantly different (*p* < 0.05).

**Table 4:** Color parameters of Atlantic horse mackerel burgers coated with different film formulations (control without coating film, chitosan, sea fennel plant (SFP) incorporated in a chitosan-based film and sea fennel extract (SFE) incorporated in a chitosan-based film) and stored at 4 °C for 12 days.

L*				
Treatment/Storage	0	4	8	12
Control	^A^ 45.35 ± 3.72 ^c^	^AB^ 48.43 ± 4.28 ^c^	^A^ 45.44 ± 4.44 ^c^	^B^ 49.53 ± 2.27 ^d^
Chitosan	^A^ 44.50 ± 2.48 ^c^	^AB^ 45.88 ± 2.74 ^c^	^AB^ 46.29 ± 2.55 ^c^	^B^ 46.99 ± 1.82 ^c^
SFP	^A^ 31.52 ± 5.28 ^a^	^A^ 32.60 ± 4.32 ^a^	^A^ 33.40 ± 2.80 ^a^	^A^ 31.83 ± 1.83 ^a^
SFE	^A^ 35.18 ± 2.86 ^b^	^AB^ 36.88 ± 2.83 ^b^	^BC^ 38.91 ± 2.93 ^b^	^C^ 39.77 ± 2.44 ^b^
a*				
Treatment/Storage	0	4	8	12
Control	^D^ 0.80 ± 0.04 ^b^	^A^ −0.32 ± 0.01 ^a^	^C^ 0.00 ± 0.00 ^b^	^B^ −0.17 ± 0.00 ^a^
Chitosan	^B^ 0.38 ± 0.06 ^a^	^A^ −0.69 ± 0.31 ^a^	^A^ −0.63 ± 0.44 ^a^	^A^ −0.38 ± 0.29 ^a^
SFP	^B^ 0.99 ± 0.14 ^b^	^A^ 0.64 ± 0.11 ^b^	^AB^ 0.84 ± 0.11 ^c^	^C^ 1.32 ± 0.04 ^b^
SFE	^A^ 2.63 ± 0.70 ^c^	^A^ 1.68 ± 0.66 ^c^	^A^ 2.54 ± 0.67 ^d^	^A^ 2.70 ± 0.50 ^c^
b*				
Treatment/Storage	0	4	8	12
Control	^A^ 7.68 ± 0.50 ^a^	^A^ 8.25 ± 0.62 ^a^	^A^ 7.14 ± 0.48 ^a^	^A^ 8.39 ± 0.54 ^a^
Chitosan	^A^ 8.42 ± 0.60 ^a^	^A^ 8.90 ± 0.70 ^a^	^B^ 10.44 ± 0.60 ^c^	^B^ 10.89 ± 0.54 ^b^
SFP	^B^ 10.95 ± 0.12 ^b^	^AB^ 9.57 ± 0.11 ^a^	^B^ 8.86 ± 0.10 ^b^	^B^ 8.86 ± 0.10 ^a^
SFE	^A^ 13.02 ± 0.16 ^c^	^A^ 13.71 ± 0.14 ^b^	^A^ 14.95 ± 0.14 ^d^	^A^ 14.06 ± 0.90 ^c^
**Hue**				
Treatment/Storage	0	4	8	12
Control	^D^ 1.45 ± 0.09 ^b^	^A^ −1.49 ± 0.02 ^a^	^C^ 0.02 ± 0.00 ^a^	^B^ −0.92 ± 0.04 ^a^
Chitosan	^C^ 1.51 ± 0.04 ^b^	^A^ −1.49 ± 0.05 ^a^	^A^ −1.50 ± 0.02 ^a^	^B^ −0.90 ± 0.01 ^a^
SFP	^A^ 1.23 ± 0.04 ^a^	^B^ 1.50 ± 0.03 ^b^	^B^ 1.47 ± 0.04 ^b^	^B^ 1.42 ± 0.02 ^b^
SFE	^A^ 1.36 ± 0.05 ^b^	^A^ 1.44 ± 0.05 ^b^	^A^ 1.40 ± 0.03 ^b^	^A^ 1.38 ± 0.01 ^a^
**Chroma**				
Treatment/Storage	0	4	8	12
Control	^A^ 7.80 ± 1.04 ^a^	^A^ 8.27 ± 0.30 ^a^	^A^ 7.15 ± 0.36 ^a^	^A^ 8.42 ± 0.76 ^a^
Chitosan	^A^ 8.29 ± 0.46 ^a^	^A^ 8.92 ± 0.62 ^a^	^B^ 10.46 ± 0.26 ^b^	^B^ 10.90 ± 0.48 ^b^
SFP	^B^ 11.00 ± 0.47 ^b^	^A^ 9.60 ± 0.48 ^a^	^B^ 8.90 ± 0.62 ^a^	^B^ 8.97 ± 0.11 ^a^
SFE	^A^ 13.29 ± 2.55 ^b^	^A^ 13.83 ± 0.13 ^b^	^A^ 15.17 ± 0.59 ^c^	^B^ 14.32 ± 0.08 ^c^

Values (mean ± standard deviation, *n* = 3) followed by a different uppercase letter in the same row are significantly different (*p* < 0.05). Values (mean ± standard deviation, *n* = 3) followed by a different lowercase letter in the same column are significantly different (*p* < 0.05).

## Supplementary Materials

The following are available online, Figure S1: Ranking test at the beginning (Day 1-I) and at the end (Day 12-II) in Atlantic horse mackerel burgers coated with different film formulations (control without coating film, chitosan, sea fennel plant (SFP) incorporated in a chitosan-based film and sea fennel extract (SFE) incorporated in a chitosan-based film) and stored at 4 °C for 12 days. Odor intensity, off-odors, color, drip loss and general acceptability were evaluated. Numbers indicate order of the samples due to treatment and for each attribute evaluated.
