# Supplementary material for: Use of Sea Fennel as a Natural Ingredient of Edible Films for Extending the Shelf Life of Fresh Fish Burgers
Source: Molecules. 2020 Nov 11;25(22):5260. doi: 10.3390/molecules25225260 (PMC7696230; doi:10.3390/molecules25225260)

Supplementary Figure 1: Ranking test (from 1 to 4) at the beginning (Day 1-I) and at the end (Day 12-II) in Atlantic horse mackerel burgers coated with different film formulations (Control- without coating film, Chitosan, Sea fennel Plant (SFP) incorporated in a chitosan-based film and Sea Fennel Extract (SFE) incorporated in a chitosan-based film) and stored at 4 ºC for 12 days. Odour intensity, Off-odours, colour, drip loss and general acceptability were evaluated.

I

II

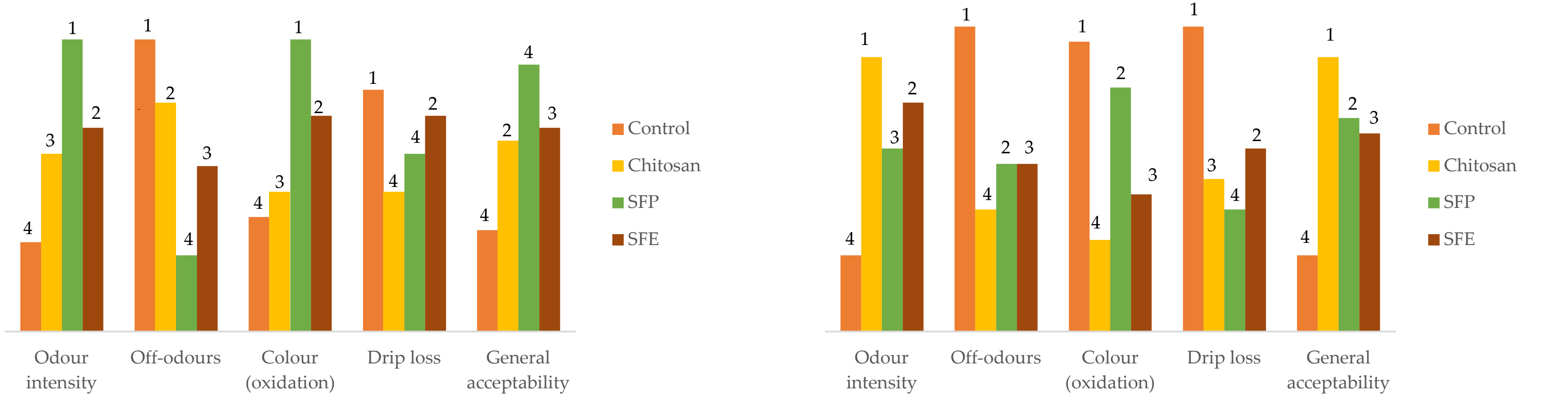

Supplement: Supplementary file 1 [file molecules-25-05260-s001.pdf]
